# Small bowel volvulus (SBV) in Northcentral Ethiopia

**DOI:** 10.1186/s12893-020-00886-6

**Published:** 2020-10-02

**Authors:** Atalel Fentahun Awedew, Woldemariam Beka Belay, Bedemariam Tadesse Amsalu, Dawit Zerihun Yalewu

**Affiliations:** 1 Department of Surgery, Addis Ababa University, Addis Ababa, Ethiopia; 2 Department of Surgery, Debre tabor Hospital, Debre Tabor, Ethiopia; 3 Department of Surgery, Debre Tabor University, Debre Tabor, Ethiopia

**Keywords:** Small bowel volvulus, Small bowel obstruction, Ethiopia, Debre Tabor

## Abstract

**Background:**

Small bowel volvulus (SBV) is a benign gastrointestinal surgical condition in which there is a torsion of all or parts of a segment of small bowel on its mesenteric axis. It has been contributed significant burden of surgical emergency as cause of small bowel obstruction in developing countries. The main objective this study was to explore clinical and epidemiological profile of Small bowel volvulus in Northcentral Ethiopia.

**Method:**

The study was conducted at Debre Tabor General Hospital, South Gondar in Northcentral Ethiopia. The medical records of patients with a discharge diagnosis of small bowel volvulus were reviewed. The patients were seen for *a 4-year* period from Jan1, 2016-Dec31, 2019. The study was hospital-based retrospective cross-sectional and data were collected with a standardized structure questioner tool. The collected data checked for any inconsistency, code, and enter *SPSS version 23* for data processing and analysis. Descriptive analyses were represented as frequency, percent, mean ± standard deviation for normal distribution, and Median ± Interquartile range for skewed data. Cross tabulation analysis was done for risk factors contributed for mortality and morbidity of SBV.

**Result:**

There were 148 patients with Small bowel volvulus as a cause of small bowel obstruction was admitted within 4 years. The annual occurrence rate was 35cases per year*. It* represented 59% of small bowel obstruction and 36.3% of bowel obstruction. The majority were males (*N* = 125, 84.5%) and females were (*N* = 23, 15.5%). The age ranged from 15 to 78 years. The mean age was 41.14 ± (SD = 15.4). The most common clinical presentation was abdominal pain 98% and vomiting 91.2%. The median time of hospital stay was 5 days (± IQR = 2). The mean duration of illness before hospital admission was 2 days (± SD = 1.4) and median was 1 day (± IQR = 2). The morbidity rate was 5.4% (*N* = 8) and the mortality rate was 3.4%(*N* = 5).

**Conclusion:**

Annual occurrence of SBV was 37 cases per year. The prevalence of SBV was 59% of small bowel obstruction and 36.3% of bowel obstruction respectively.

## Background

The origin of the word volvulus is derived from the Latin word “volvere” which means turn or roll [1]. Small bowel volvulus (SBV) is a benign gastrointestinal surgical condition in which there is a torsion of all or parts of a segment of small bowel on its mesenteric axis [2]. Small bowel volvulus is a rare cause of surgical emergency in the western and the USA while it is commonly found in Africa, India, and Iran [3]. Small bowel volvulus can be Neonatorm volvulus, primary or secondary according to the cause. The mechanism and underlying cause of primary SBV has not been well illustrated but the presumed factors include unusual mobility of small bowel, laxity of abdominal wall, physical exercise, absence of mesenteric fat, constipation or diarrhea, and the unusual length of small bowel segment [2–5]. Secondary SBV occurs in the presence of acquired underlying conditions such as post-op adhesion, tumors, pregnancy, diverticular disease, and bands [2] while Neonatorm volvulus is due to malrotation of bowel and defective fixation of mesentery [3]. Small bowel volvulus is a rare cause of mechanical small bowel obstruction. The incidence rate of SBV is high (24-60per 100,000populations) in Africa, Asia and Middle Eastern while low (1.7–5.7 per 100,000populations) in western [6]. The overall mortality rate of SBV varies from 10 to 38%, viable bowel 3–13% in western and 20–25% in Africa and Indian. The mortality rate of gangrenous bowel has been reported in a range of 20–100% [7].

## Methods

### Study area, period and design

The study was conducted at Debre Tabor General Hospital, South Gondar in Northcentral Ethiopia. The medical records of patients with a discharge diagnosis of small bowel volvulus were reviewed. The patients were seen for a 4-year period from Jan1, 2016-Dec31, 2019. The study was hospital-based retrospective cross-sectional and data were collected with a standardized structure questioner tool. The Question tool contained demography, clinical presentation, and duration of illness, procedures and intraoperative findings, postoperative complications, and management outcome. All patients from age 15 years and above with clinical or/and radiological diagnosis of Small bowel volvulus which managed with conservatively and /or operatively or died due to confirmed Small bowel volvulus was included while paralytic Ileus (functional obstruction) and age below 15 years were excluded from the study. The hospital provides clinical service for approximately 900 inpatient and 80,000 outpatients in a year that comes from the catchment area of the population around 3.5 million.

### Data collection and analysis

Data collected with a structured questionnaire that is developed by the English language and translated to the Amharic language. The tool was prepared after reviewed different literature and pretested before data collection. The collected data checked for any inconsistency, code, and enter SPSS version 23 for data processing and analysis. Normal distribution was evaluated with the Q-Q plot test and histogram diagram. Descriptive analyses were represented as frequency, percent; mean ± standard deviation for normal distribution, and median ± interquartile range for skewed data. Cross tabulation analysis was done for risk factors contributed for mortality and morbidity of SBV.

### Ethical consideration

Ethical Review Committee of Debretabor University was approved and collection started after approval of the proposal. The confidentiality and privacy of patients maintained during data collection. The data collected retrospectively from the medical chart and consent from patients is not needed

## Results

There were 148 patients with small bowel volvulus as a cause of small bowel obstruction was admitted within 4 years. It represented 59% of small bowel obstruction and 36.3% of bowel obstruction. The majority of SBV were males (*N* = 125, 84.5%) and females were (*N* = 23, 15.5%). The majority of SBV patients were rural residents (*N* = 121, 81.8%) and urban residents were (*N* = 27, 18.2%).The age ranged from 15 to 78 years. The mean age was 41.14 years ± (SD = 15.4) (Table 1).

**Table 1:** The Demographic and Characteristics of the Patients ***Small bowel volvulus*** in Debre Tabor Hospital, Northcentral Ethiopia, 2020

***Item***		***Frequency(N)***	***Proportion (%)***
***Age*** ***N = 148***	***15–25***	28	18.9
	***26–35***	34	23.0
	***36–45***	33	22.3
	***46–55***	29	19.6
	***56–65***	15	10.1
	***> 66***	9	6.1
***Sex*** ***N = 148***	***M***	125	84.5
	***Female***	23	15.5
***Residence*** ***N = 148***	***Rural***	121	81.8
	***Urban***	27	18.2

The most common clinical presentation was abdominal pain 98% and vomiting 91.2%. Most of the patients presented within 48 h (67.6%) and 48-72 h (19.6%). The mean of hospital stay was 5.3 days (± SD = 2.9) and ranged from*1–18 days*. The median time of hospital stay was 5 days (± IQR = 2). The mean duration of illness before hospital admission was 2 days (± SD = 1.4) and median was 1 day (± IQR = 2). Majority of the patient respond for conservative treatment with NPO, NGT decompression and fluid resuscitation (43.2%). The most common procedure for non-respond for conservative measures were *derotation and milking (DAM)* for 39.9% and primary resection and anastomosis for 16.2%(Table 2). The overall morbidity rate was 5.4% (*N* = 8) and the mortality rate was 3.4%(*N* = 5) (Table 2 & 3). Wound infection was found as most complication. *The morbidity rate for patients who had undergone surgery was 9.6% and the mortality rate was 6%.*

**Table 2:** The Clinical presentation of Patients with Small bowel volvulus in **D**ebre Tabor Hospital, Northcentral Ethiopia, 2020

***Item***		***Frequency(N)***	***Proportion(%)***
***Clinical Symptoms***	***Abdominal Pain***	145	98
	***Vomiting***	135	91.2
	***Abdominal Distention***	91	61.5
	***Constipation***	100	67.6
***Clinical Sign***	***Drainage Vital sign***	19	12.8
	***Abdominal Tenderness***	126	85.1
	***Hypo/Hyperactive bowel sound***	71	48
	***Visible peristalsis***	96	64.9
	***Hyper tympanic***	123	83.1
***Mode Treatment***	***Emergency***	83	56.1
	***Conservative***	64	43.2
	***Death before procedure***	1	0.7
***Procedure***	***Derotation and Milking***	59	39.9
	***Primary resection and anastomosis***	24	16.2
	***Conservative***	64	43.2
	***Death before procedure***	1	0.7

**Table 3:** The Pattern and outcomes of Patients with Small bowel volvulus in Debre Tabor Hospital, Northcentral Ethiopia, 2020

***Item***		***Frequency(N)***	***Proportion(%)***
***SV***	***Simple SBV***	123	83.1
	***Gangrenous SBV***	25	16.9
***Type of SBV***	***Primary***	148	100
	***Secondary***	0	0
***Outcome***	Improved	132	89.2
	Morbidity	8	5.4
	Leave against medical advice	2	1.4
	Mortality	5	3.4
***Duration of illness before hospital visits***	< 48 h	100	67.6
	48-72 h	29	19.6
	72-96 h	8	5.4
	> 96 h	11	7.4
***Hospital stay*** ***N = 148***	<=7 days	123	83.1
	> 7 days	25	16.9
***Morbidity*** ***N = 8***	Wound Infection	4	2.7
	Anastomosis Leak	1	0.7
	Fistula	1	0.7
	Paralytic illness	1	0.7
	others	1	0.7

Cross tabulation analysis showed morbidity rate of SBV was more likely female 8.7%vs4.8%, common in age > 40 years 5.8%vs5.1%, urban 7.4%vs. 5%, Hospital stay > 7 days 24%vs1.6%, patients presented 72–96 h. 12.5%,as compared less than 48 h 5%, gangrenous SBV 20%vs.2.4%,emergency procedure 9.6% vs0% conservative and primary resection and analysis16%vs derotation and milking (DAM).

Cross tabulation analysis showed mortality of SBV was more likely common in age < 40 years 3.8%vs2.9%, urban 3.7%vs.3.3%, hospital stay > 7 days 4%vs3.3%, patients presented above 97 h 18.2% as compared less than 48 h 2%, gangrenous SBV 8%vs.2.4%,emergency procedure 6% vs0% conservative and derotation and milking (DAM)7%vs.4% in primary resection and analysis.

## Discussion

Small bowel volvulus is a rare cause of small bowel obstruction in the western and the USA [1, 2, 8]. The incidence rate of SBV is high (24–60 per 100,000populations) in Africa, Asia and Middle Eastern while low (1.7–5.7 per 100,000populations) in western [6]. Annual occurrence in the USA and Europe is quite low compared with that in Africa, Middle East countries, and Asian [2, 3, 7, 9–11] (Table 4). A 57-years data review in the USA found that annual occurrences of SBV was 0.6case per year and SBV accounted for 6.9% of all bowel obstruction [7]. Welch CE 1958 reported that the prevalence reduction from 6.9 to 3.3% in the period of 1930–1958 in the USA [12] .A. Roggo and L. W. Ottinger (1992) at the Massachusetts General Hospital over 10 years reported those 35 small bowel volvulus were treated which represented 1.7% of bowel obstructions and 4% of small bowel obstructions [7]. Vaez-Zadeh et al. (1969) reported the incidence of small bowel volvulus in Iran was 19.6 and 41% in bowel obstruction and small bowel obstruction respectively [2].

**Table 4:** Literature Review

Authors	Atalel F. Current study	M. Demssie	Roggo	Frazee	Welchi	Veaz-Zadah
Study Place	DGH, Ethiopia	Southern, Ethiopia	Bosten	Rochester MN	Glasgow UK	Iran
Study period	Jan1,2016-Dec31, 2019	June 1992toMay 1996	1980–1990	1975–1984	1965–1980	1962–1965
Duration Years	4	4	10	10	15	5
No of patients	148	98	35	57	53	41
Annual occurs	37	24.5	3.5	5.7	3.5	8.2
Male to Female	5.4:1	8.8:1	1:1.2	1:1	1:1.12	9.25:1
Mean age	41.14	34.1	67	59	54	42
Primary SBV(%	100	94.9	14	11	30	80
Secondary SBV(*%	0	5.1	86	89	70	20
Gangrenous SBV(%	16.9	28.7	46	49	43	–
Overall Mortality	3.4	13.3	9	12	28	15
Viable SBV	2.4	8.3	0	3	11	–
Gangrenous SBV	8	25.9	17	21	47	–

A series of research done in Ethiopia reported that small bowel volvulus accounted sizable portion of bowel obstruction; Tegegne A. 1992(18.3%) [13], Lintjorn B.1981et al (38.6%) [14], M. Demissie 2001(41.7%) [2], Gizaw TA.et al. 2016, (36.4%) [15], Soressa U.et al. 2016, (30.3%) [16]. I.O.McDonald et al. 1980 reported more than 50% of bowel obstruction due to Small bowel volvulus in Nepal [5]. This great discrepancy of the prevalence of small bowel volvulus may due to dietary factors that when a bulky bolus of food entered the proximal jejunum, that loop felt down into pelvis and causing empty distal bowel to rise into a right upper quadrant. Rapid emptying of the stomach and diaphragm makes the distal bowel spread across the left upper quadrant. As this distal loop themselves filed up would fall into left quadrat completing 360^0^ twists [3]. Primary SBV is more common in developing countries while secondary SBV is more in developed countries. Frazee RC et.al 1988 (UK) and A. Roggo and L. W. Ottinger 1992(USA) noted that secondary SBV accounted for 89%&86% of SBV respectively [7, 9] (Table 4). A Research done by Vaez-Zadeh et al. 1969(Iran), M. Demssie 2001(Ethiopia) and De Souza LJ1976 (Uganda) showed that primary SBV accounted 80, 94.9 and 100% of SBV respectively [2, 3, 11] (Table 4). We found that primary SBV accounted 100% of SBV. The presumed risk factors in different literature were hypermobility of bowel gut, hypermobility of mesentery, and bulky and poorly digestible diet taken at long intervals [2, 7, 9, 17].

The speculation was some populations have long mesenteric and short roots of mesentery which would allow abnormal mobility of whole or segment of the small bowel. Diet and pattern of feeding is also the most relevant risk factor for occurrences of primary SBV. The Abrupt change of dietary habit of ingestion of a single large amount and bulky food after a long interval of fasting or empty of bowel may induce forceful bowel peristalsis, resulting in SBV [2, 7, 9, 17]. Secondary small bowel volvulus occurs in the presence of underlying identified predisposed conditions. It is mainly seen in bands, post-op adhesion, malrotation, or midgut nonrotation in newborn and young children and a gastrointestinal infestation of the parasite. The prevalence of secondary SBV is more common in western, constituting a 70–90%of case [7].

The prevalence of primary SBV is more common in children and young while secondary SBV is dominantly in old age. A study conducted in the different areas stated that the mean age was 67 years in the USA,34 years in Ethiopia, 42 years in Iran,59 years in the UK (2,3,7,13] (Table 4). We found that the mean age was 42 years. The incidence of SBV is a male predominance, particularly in developing countries. The research described that male to female proportion 1:1.2 in USA, 1:1.12 in UK, 9.25:1 in Iran, 8.8:1 in Ethiopia [2, 3, 7, 9]. We found similar results on the male to female proportion 5.4:1(Table 4). The exact cause of sex discrepancy is not well-illustrated but possible reasons suggested that spacious female pelvic area allowed a greater possibility of spontaneous reduction of volvulus, females have a lax abdominal wall due to childbearing, doing less heavy and prolonged exercise and more obese [2, 3, 7]. Our finding noted that SBV was more common *in rural resident* due to more childbearing, doing less heavy, long time fasting and higher fiber diet.

The literature review summarized the common complaints of SBV abdominal pain 94–100%, vomiting 83–100%, and abdominal distention 55–100% [7]. Our result identified the common clinical complaints of SBV similar to the previous review; abdominal pain 98%, vomiting 92.1% and distention 61.5% and frequent physical findings were abdominal tenderness 85.1% and visible peristalsis 64.9%.

SBV has been contributing a significant burden in mortality and morbidity especially when the bowel becomes gangrenous, ischemic, and develops peritonitis. The prevalence of gangrenous SBV depends on the health-seeking behavior of the community, access to a health facility, early diagnosis, and economy of the patients. The incidence of gangrenous SBV was 28.7 in Ethiopia, 46% in the USA, and 43% in the UK [2, 7, 9].

Our study found that the incidence of gangrenous SBV 16.9%. This difference study conducted in the USA and UK was in the early 1950s and currently increases access to surgical investigation modality decreasing the incidence of gangrenous SBV.

SBV had a high mortality rate but currently mortality due to SBV decreasing due to the advance of surgical health, investigation, and early diagnosis. Overall mortality rate reported 26% in Nepal,13.3% in Ethiopia,28% in UK,9% in USA and 15% in Iran [2, 3, 5, 7, 9]. Our finding was lowered compared with the previous study (3.4%).

## Conclusion

Annual occurrence of SBV was 37 cases per year. The prevalence of SBV was 59% of small bowel obstruction and 36.3% of bowel obstruction respectively. It had low morbidity and mortality rate (5.4 &3.4% respectively). The majority of SBV were respond with conservative treatment with NPO, NGT decompression and resuscitation (43.2%).

## Data Availability

The datasets used and/or analyzed during the current study available from corresponding author on reasonable request.

## Abbreviations

COR: Crude Odd Ratio
DAM: Derotation and Milking
IQR: Inter Quartile Range
NGT: Nasogastric tube
NPO: Nill per os
OR: Odd Ratio
SBO: Small bowel obstruction
SBV: Small Bowel volvulus
SD: Standard deviation
SPSS: Statistical package for Social Science
UK: United Kingdom
USA: United States of America
